# An uncommon case of cutaneous collagenous vasculopathy

**DOI:** 10.1016/j.jdcr.2026.01.044

**Published:** 2026-02-03

**Authors:** Trina Nguyen, Lindsey Mohney, Kayley Erickson, Elaine Kunzler

**Affiliations:** a Department of Dermatology, Case Western Reserve University School of Medicine, Cleveland, Ohio; b Department of Dermatology, University Hospitals, Cleveland, Ohio; c Department of Veterans Affairs Louis Stokes Medical Center, Dermatology Service, Cleveland, Ohio

**Keywords:** cutaneous collagenous vasculopathy, superficial microangiopathy, telangiectasia, vasculopathy

## Introduction

Cutaneous collagenous vasculopathy (CCV) is a rare, idiopathic superficial microangiopathy first characterized in 2000 by Salama and Rosenthal.^1^ There have been fewer than 200 cases documented since CCV was first described, though CCV may be underdiagnosed due to its clinical similarity to other vascular pathologies.^2^ Hence, there is still a significant gap in CCV knowledge. In contrast to other telangiectatic processes, CCV is characterized histologically by dilated vascular structures that contain deposits of hyaline material within the vessel walls.^3^ This condition has been documented in both men and women and appears more commonly in fair-skinned, middle-aged to older adults; though pediatric cases have also been described.^2^, ^3^, ^4^, ^5^ Clinically, individuals present with diffuse telangiectasias, initially on the lower extremities, that progressively spread to the trunk and upper extremities.^1^, ^2^, ^3^, ^4^ The telangiectasias are generally asymptomatic, though some patients report pruritus and pain.^4^

While the exact pathophysiology remains unclear, CCV has been observed more frequently in individuals with underlying medical conditions, such as hypertension and type 2 diabetes mellitus.^6^ We present a case of CCV in an adult Caucasian male Fitzpatrick skin type I with a history of psoriasis and obesity. This case contributes to existing literature by illustrating early-onset CCV in a patient without common comorbidities reported in association with this condition and by exploring efficacious treatment options.

## Case report

**Fig 1:**
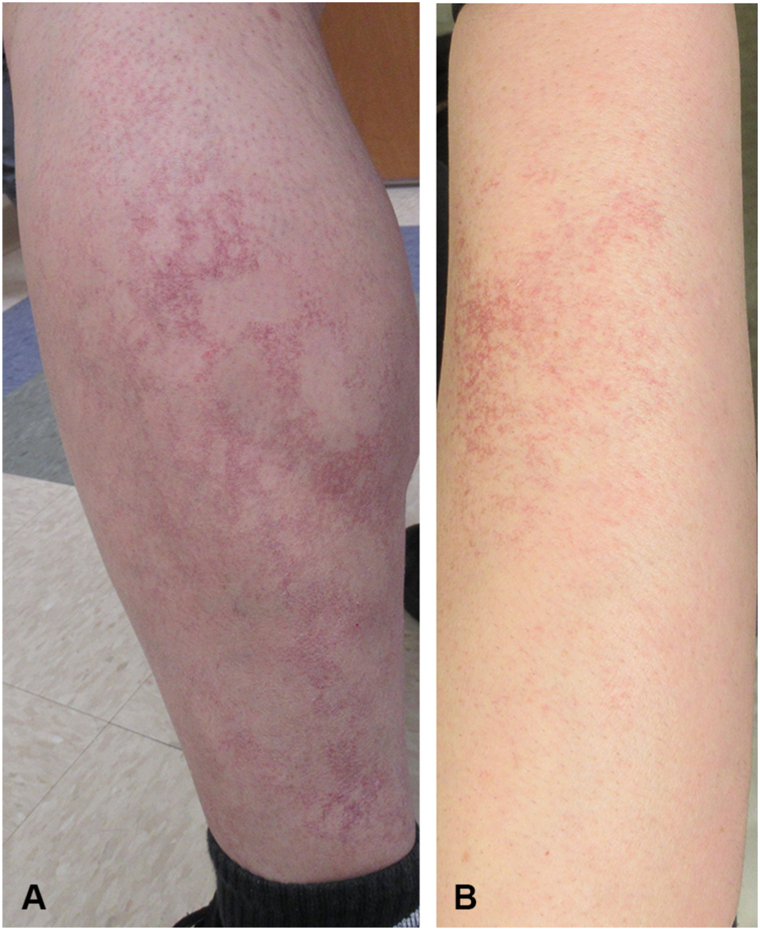
Clinical presentation of CCV at first office visit. **A,** Telangiectatic, reticular patches on the left lower leg. **B,** Telangiectatic macules coalescing into patches on the right forearm. *CCV*, Cutaneous collagenous vasculopathy.

A 33-year-old Caucasian male with a history of psoriasis presented to our dermatology clinic with a 10-year history of asymptomatic, progressively spreading telangiectasias. On examination, he exhibited telangiectatic macules coalescing into reticular patches on the forearms, thighs, and lower legs (Fig 1, *A* and *B*). Given lack of systemic symptoms, absence of inheritance pattern, and no evidence of bleeding diathesis, the clinical impression at that time included generalized essential telangiectasia, livedo racemosa, and telangiectasia macularis eruptiva perstans. Upon reevaluation 5 months later, the patient had progression of his lesions with continued sparing of the trunk. Two punch biopsies from the left anterior medial thigh were collected, 1 from an uninvolved central area and the other from involved skin. This method was used for comparison and to catch potential thromboses that can be found on biopsy in central/uninvolved skin of livedo racemosa.

**Fig 2:**
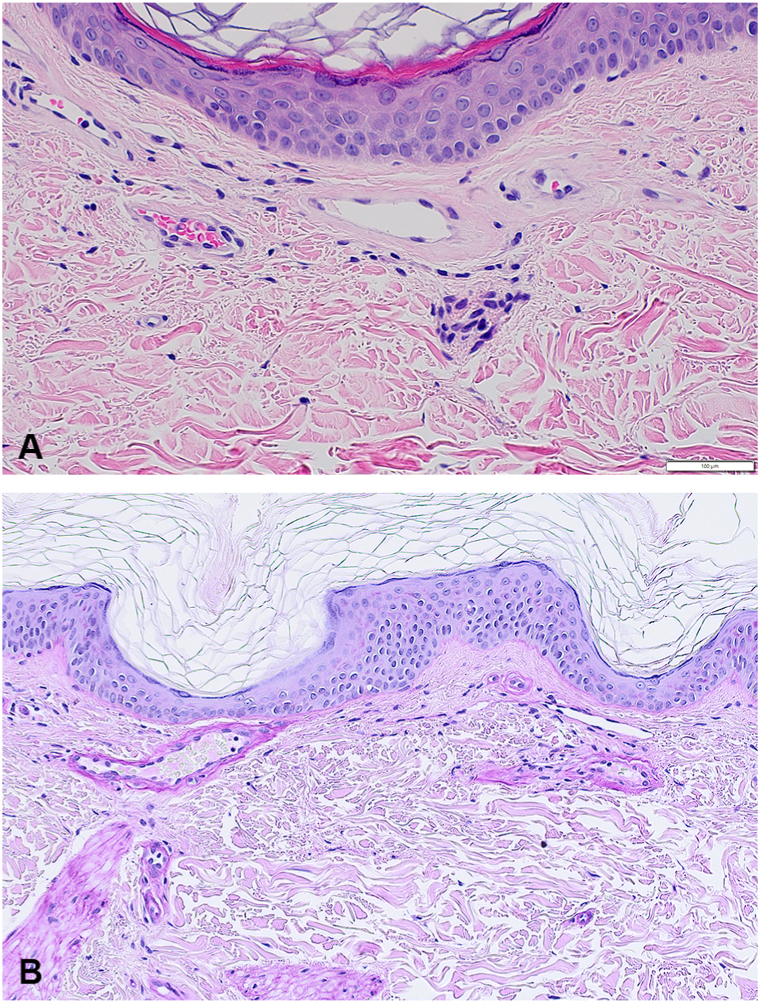
Histopathology examination from punch biopsy. **A,** Hematoxylin and eosin stain at 400× magnification demonstrating homogenous *pink* material surrounding superficial blood vessels. **B,** Periodic Acid-Schiff (PAS) stain at 200× magnification highlights the thickened, hyaline blood vessel walls.

**Fig 3:**
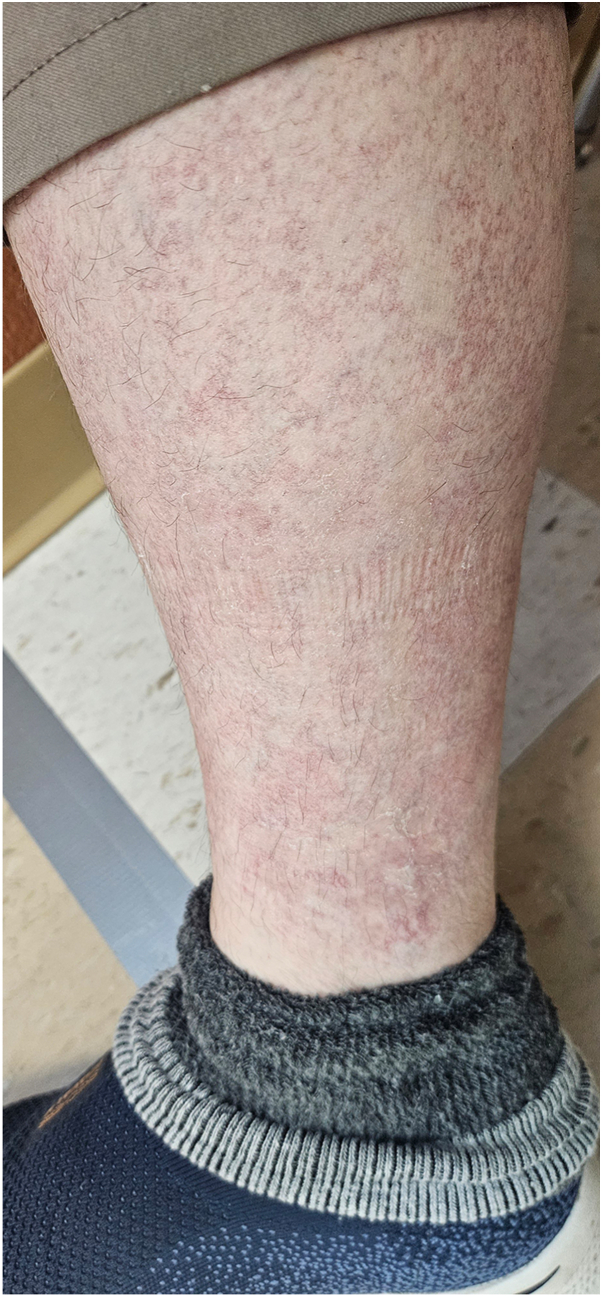
Photograph of the right lower leg taken 1-month post-treatment demonstrating decreased telangiectatic macules and patches following 1 session of pulsed dye laser (595-nm).

Histopathologic examination from both sites revealed dilated superficial blood vessels with thickened hyaline walls highlighted by Periodic Acid-Schiff stain (Fig 2, *A* and *B*). CD117 immunohistochemical stain demonstrated a normal number of mast cells. There were no significant differences between the 2 punch biopsies. These findings were consistent with cutaneous collagenous vasculopathy. The patient was initiated with treatment by pulsed dye laser (PDL, 595 nm). As of the time of this writing, the patient has completed 2 sessions (pulse width-3 ms, energy 7 mJ/cm2, spot size: 10 mm, endpoint: vessel disappearance). The patient has experienced marked clinical improvement and will continue treatments (Fig 3). The post-treatment photograph was obtained 1 month after the first treatment.

## Discussion

Cutaneous collagenous vasculopathy (CCV) is a rare idiopathic microangiopathy characterized by diffuse, progressive telangiectasias.^1^^,^^3^ Because of its rarity, there has not yet been predilection of CCV to any ethnic groups. Although most patients are middle-aged or older, pediatric and early adult-onset cases have been reported.^5^ This patient’s disease onset at approximately 23 years of age adds to the growing evidence that CCV can occur earlier than previously recognized.^6^ The present case also contributes to the expanding clinical spectrum of CCV by illustrating its occurrence in a younger adult.

Previous studies have proposed a potential relationship between CCV and underlying systemic conditions, most notably hypertension and type 2 diabetes mellitus.^6^ Our patient’s comorbidities included obesity and psoriasis which can be associated with other metabolic disorders. Although the exact pathogenesis is unknown, hypotheses include microvascular injury, glycoprotein deposition, and alterations in extracellular matrix remodeling leading to hyalinization of vessel walls.^2^ Additionally, the patient’s history of psoriasis raises questions about whether chronic cutaneous inflammation or microvascular changes associated with psoriatic disease could play a contributory role, though current evidence remains insufficient to establish such a connection and warrant cautious interpretation. Presence of CCV with comorbid psoriasis has rarely been reported, and no clear pathogenic link has been established.^7^

Several treatment modalities have been used in CCV. Pulsed dye laser (PDL) has been used successfully for patients.^8^^,^^9^ In 2012, Escheverria and Botella-Estrada conducted laser therapy with 585 nm pulsed dye laser on a patient’s legs for her CCV. When blanching was obtained with this treatment modality, it was then applied to the rest of her body.^8^ In 2015, Basso et al treated an elderly patient presenting with an extensive form of CCV involving the trunk, upper and lower limbs with Multiplex PDL 595-nm/Nd: YAG 1064-nm laser and optimized pulsed light. This approach resulted in almost complete clearance of the widespread lesions.^9^

In addition to PDL, several medical therapies used in related vascular disorders have been explored, including beta-blockers, vitamin C, and rutin.^10^ McFeeters et al recently reported clinical improvement in a patient with CCV treated with intense pulsed light in conjunction with medical therapy.^10^ The patient initially received vitamin C 500 mg and rutin 50 mg twice daily with limited response. Propranolol 20 mg daily was subsequently added, followed by a series of 5 intense pulsed light treatments. The patient experienced both improved lesion appearance and reduced disease progression, prompting an increase in propranolol to 40 mg 3 times daily. Such multimodal approaches may help guide management when access to laser therapy is limited or when combined therapeutic strategies are preferred.

These findings underscore the importance of recognizing CCV across a broader age range and in patients without typical comorbidities. Furthermore, this case adds to the growing evidence supporting pulsed dye laser as an effective therapeutic option, highlighting its role in achieving meaningful and sustained improvement. Overall, this case highlights the relevance of histopathologic confirmation in suspected telangiectatic disorders and expands the spectrum of recognized CCV presentations.

### Declaration of generative AI and AI-assisted technologies in the writing process

AI was used for language editing only. All content and ideas belong 100% to the authors.

## Conflicts of interest

None disclosed.
